# Whole genome sequencing analyses revealed that *Salmonella enterica* serovar Dublin strains from Brazil belonged to two predominant clades

**DOI:** 10.1038/s41598-022-14492-4

**Published:** 2022-06-22

**Authors:** Fábio Campioni, Felipe Pinheiro Vilela, Guojie Cao, George Kastanis, Dália dos Prazeres Rodrigues, Renata Garcia Costa, Monique Ribeiro Tiba-Casas, Lanlan Yin, Marc Allard, Juliana Pfrimer Falcão

**Affiliations:** 1 Departamento de Análises Clínicas, Toxicológicas e Bromatológicas, Faculdade de Ciências Farmacêuticas de Ribeirão Preto - USP, Av. do Café, s/n. Bloco S – Sala 41, Ribeirão Preto, SP 14040-903 Brazil; 2 Division of Microbiology, Office of Regulatory Science, Center for Food Safety and Applied Nutrition, U.S. Food and Drug Administration, College Park, MD USA; 3 Laboratório de Enterobactérias, FIOCRUZ/Fundação Instituto Oswaldo Cruz, Avenida Brasil, 4365, Pavilhão Rocha Lima, 3°andar, Manguinhos, Rio de Janeiro, RJ Brazil; 4 Centro de Bacteriologia, Instituto Adolfo Lutz, Av. Dr. Arnaldo, 351, São Paulo, SP Brazil; 5 Office of Analytics and Outreach, Center for Food Safety and Applied Nutrition, U.S. Food and Drug Administration, 5001 Campus Drive, College Park, MD USA

**Keywords:** Microbiology, Bacteria

## Abstract

*Salmonella* Dublin is a cattle-associated serovar sporadically causing disease in humans. *S.* Dublin strains isolated in Brazil and in other countries were analyzed to determine their phylogenetic relationships, the presence of genes, plasmids, genomic regions related to virulence and antimicrobial resistance genes repertoire, using WGS analyses. Illumina was used to sequence the genome of 112 *S.* Dublin strains isolated in Brazil from humans (n = 82) and animals (n = 30) between 1983 and 2016. Furthermore, 87 strains from other countries were analyzed. WGSNP analysis revealed three different clades, in which the strains from Brazil belonged to two clades, A and C. Most of the genes and genomic regions searched varied among the strains studied. The siderophore genes *iroB* and *iroC* were exclusively found in strains from Brazil and *pegD* gene, related to fimbrial adherence determinants, were positive in 124 strains from clades A and B but absent in all the strains from clade C (n = 71). Eleven plasmid replicons were found in the strains from Brazil, and nine were exclusively found in strains from other countries. The antimicrobial resistance genes *mdsA* and *mdsB,* that encode an efflux pump, were found in all the strains studied. The strains from Brazil carried other resistance genes, such as *tet(A)* (n = 11), *tet(B)* (n = 4) and *tet(C)* (n = 4), *blaTEM-1* (n = 4), *catA1* (n = 1), *aadA1* (n = 1), and *sul1* (n = 1). In conclusion, *S.* Dublin strains isolated in Brazil presented some few unique genes not found in strains from other countries and were allocated into two distinct clades with strains of human and animal origin epidemiologically related. This fact stresses the zoonotic potential of *S.* Dublin circulating in Brazil for more than 30 years.

## Introduction

Salmonellosis caused by *Salmonella enterica* serovars has been a major foodborne disease in many countries, where it accounts for a burden of morbidity and mortality. Non-typhoidal *S. enterica* serovars usually cause self-limiting diarrhea, but some of them have adapted to cause invasive disease and systemic infection in humans that have resulted in an estimated number of 680,000 deaths every year^1,2^.

*Salmonella enterica* serovar Dublin (*S.* Dublin) is a host-adapted cattle-associated serovar that sporadically causes disease in humans, but its incidence, antimicrobial resistance, and severity of disease have increased in recent years^3,4^. Compared with other non-typhoidal serovars, the *S.* Dublin strains are highly invasive, with invasion indices above 40% reported around the world; the infection indices of most isolated serovars, such as Typhimurium and Enteritidis, rarely exceed 5%^4–8^.

In general, *S.* Dublin causes serious infections in both bovine species and humans, and can ultimately lead to death. In bovine species, mild cases of the disease result in acute diarrhea and abortion in pregnant cows, which are sometimes associated with other clinical symptoms. Another clinical manifestation of *S.* Dublin infection is a typhoid fever-like disease in which the host generally becomes an asymptomatic chronic carrier of the microorganism and excretes it for years and, in some cases, spanning the animal’s lifetime^5,8,9^.

Human infection with *S.* Dublin is mostly caused by the consumption of contaminated cow’s milk and beef. The disease is usually severe and can be fatal to patients with underlying chronic diseases, but it can be indistinguishable from typhoid fever in some patients. Currently, the bacterial factors responsible for the invasive characteristic in humans are poorly understood^4,8–10^.

Whole genome sequencing (WGS) has been a valuable tool to study the epidemiology and population structure of this serovar around the world^1,11–14^. In Brazil, few studies on *S.* Dublin strains using molecular typing have been conducted, and only one has analyzed the diversity of the strains by CRISPR methodologies using sequenced strains^14,15^.

The present study used WGS analyses to assess the phylogenetic relationships among a set of *S.* Dublin strains isolated in Brazil between 1983 and 2016. The collected data were compared with those from publicly available strains of this serovar isolated in other countries, in order to identify possible genomic differences among the strains circulating in Brazil over a 33 year-period.

## Materials and methods

### Bacterial strains

This study included 112 *Salmonella enterica* serovar Dublin strains isolated from humans (n = 82) and animals (n = 30) in 10 states from Brazil between 1983 and 2016 (Table S1). The strains were provided by two *Salmonella* reference laboratories in Brazil, the Adolfo Lutz Institute at São Paulo (IAL-SP) and Oswaldo Cruz Foundation at Rio de Janeiro (FIOCRUZ-RJ) and were selected to represent different years, sources and places of isolation of the strains. The accession numbers of the strains studied were reported previously^16^ and included in Table S1. Furthermore, 87 *S.* Dublin genomes were downloaded and compared with the strains from Brazil studied herein, detailed information about these strains are displayed in Table S1. These strains were selected to represent the different countries and years of isolation presented in the different single nucleotide polymorphism (SNP) clusters found in the Pathogen Detection database. The SNP clusters were provided by the Pathogen Detection database accessed on May 28, 2020 and, all the *S.* Dublin clusters had some strains from different countries selected.

### Library preparation and sequencing

The libraries and runs were prepared according to Campioni et al.^16^ and the samples were sequenced at the Center for Food Safety and Applied Nutrition at the United States Food and Drug Administration (College Park, Maryland, United States of America).

### Genomic data analyses

**Figure 1:**
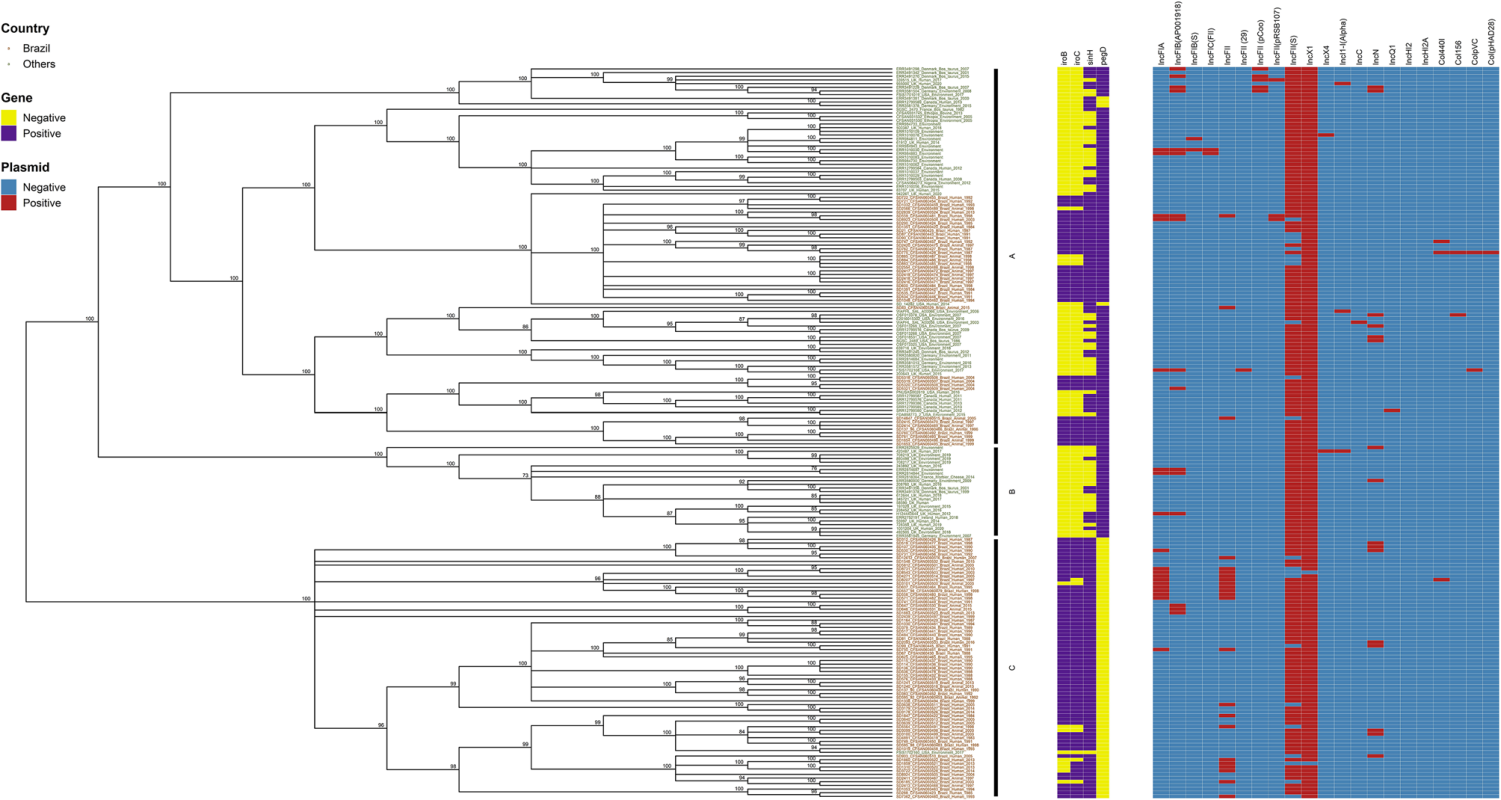
Parsimony tree based on the SNP-analysis of 112 *Salmonella enterica* serovar Dublin strains isolated in Brazil.

kSNP3.0^17^ with k value as 19 was used to construct a matrix of core SNPs. GARLI 2.0^18^ was used to construct the maximum likelihood phylogenetic tree (ratematrix = 6rate, ratehetmodel = gamma). Multiple runs were performed (n = 100) to ensure that results were consistent. To estimate support for each node, phylogenies were reconstructed for 1,000 bootstrap replicates of the data set. Python program SumTrees v4.0.0^19^ was used to generate one consensus tree with bootstrap value at a 70% threshold. Genetic algorithm approaches for the phylogenetic analysis of large biological sequence datasets under the maximum likelihood criterion. R package ggtree v3.1.3^20^ was used to visualize the phylogenetic tree and genomic analyses data in Fig. 1.

The Basic Local Alignment Search Tool (BLAST)^21^ was used to search for the presence of the following genes/genomic regions related to the *S.* Dublin invasome^1^ and region-specific clades previously described^12^: gene island from *S*. Typhimurium ST313 (ST313-GI) strain D23580 (GenBank Accession Number FN424405) and *bstA* (st313-td) gene^22^; Gifsy-2 like prophage (Accession Number: NC_010393); the two different T6SSs present in reference *S.* Dublin str. CT_02021853, including T6SSSPI-6 (Locus_tag: SeD_A0289—SeD_A0326) and T6SSSPI-19 (Locus_tag: SeD_A1212—SeD_A1243); SPI6 and SPI7; γ-glutamyl transpeptidase encoding gene (*ggt*); an outer membrane protein encoding gene (*pagN*); and the Vi coding genes *tviA*, *tviB*, *tviC*, *tviD* and *tviE*^1^.

PlasmidFinder available at the Center for Genomic Epidemiology (https://www.genomicepidemiology.org) was used to detect plasmid replicons in the strains studied^23^. The genotypic antimicrobial resistance, stress and virulence genes profiles of the 204 *S.* Dublin strains studied (Table S1) were accessed on May 28, 2020 on the publicly available NCBI Pathogen Detection database (https://www.ncbi.nlm.nih.gov/pathogens/). The analyses relied on the metadata available at the *S. enterica* database accessed on May 28, 2020. The search tool on the database was used to filter only the strains belonging to the Dublin serovar, including the 112 strains from Brazil (Table S1). All the genes searched by the Pathogen detection database are available at https://www.ncbi.nlm.nih.gov/pathogens/refgene. In addition, Virulence Factors of Pathogenic Bacteria database (http://www.mgc.ac.cn/cgi-bin/VFs/v5/main.cgi?func=VFanalyzer) was used to search for the virulence factors of the *Salmonella* genus among the strains studied. Finally, the sequence types of the strains studied based on the Achtman Multilocus Sequence Typing (MLST) scheme, was assessed by using the Enterobase database (https://enterobase.warwick.ac.uk/).

## Results

### Whole genome SNP analysis

The 199 *S.* Dublin strains were allocated into three mains clades (Fig. 1): clade A consisted of 103 strains from Brazil (n = 42), USA (n = 14), Canada (n = 9), UK (n = 8), Denmark (n = 6), Germany (n = 5), Ethiopia (n = 3), France (n = 1), Nigeria (n = 1), and some strains from unidentified countries (n = 14); clade B consisted of 25 strains isolated in UK (n = 16), Denmark (n = 2), Germany (n = 2), France (n = 1), Ireland (n = 1), and some strains from unidentified countries (n = 3); and clade C consisted of 71 strains from Brazil (n = 70) and the USA (n = 1). Specifically, the 112 *S.* Dublin strains isolated in Brazil were allocated into two predominant clades by whole genome SNP analysis (Fig. 1).

### Detection of genomic regions, genes and plasmids

A total of 246 genes, plasmids and genomic regions related to virulence were searched in the strains studied. The genes *tviA*, *tviB*, *tviC*, *tviD*, and *tviE* contained in the *viaB* operon, responsible for the Vi capsule biosynthesis, were absent in all but one strain studied, SARB13 (SGSC-2470), (Table S1). The *bstA* (st313-td) gene, ST313GI genomic island, Gifsy-2 like prophage, T6SS_SPI-6_ and T6SS_SPI-19_, SPI-6 and SPI-7, and the virulence genes *PagN* and *ggt* were found in all the strains studied. The exception was the strain SARB13, which did not carry the *bsta* gene (Table S1).

The genes *iroB* and *iroC*, related to iron uptake and defense against oxidative stress, were found in 99 and 101 strains, respectively; all of them were from Brazil and belonged to the two clades comprising strains from this country (Fig. 1). The genes *iucA*, *iucB* and *iucC*, related to aerobactin production, were detected in three strains from Brazil from clade A (n = 2) and C (n = 1). The cytolethal distending toxin related genes *cdtB* and *pltA* were found only in one strain from clade A with no country assigned (Table S1). The autotransporter related gene *tibA* were found in six strains from cluster A (5), isolated in Denmark (3) Germany (1) and the UK (1); and from cluster B (1) isolated in the UK (Table S1). The genes *pilQ*, *pilR*, *pilV* and *pilW*, related to adherence (pili), were found in three strains from cluster A (2) isolated in the USA (1) and the UK (1); and from cluster B (1) isolated in the UK (Table S1). The genes *faeC*, *faeD* and *faeE*, related to adherence (fimbriae), were found in only one strain from cluster A with no country assigned (Table S1). The *pegD* gene, related to fimbrial adherence determinants, were absent in all the strains from clade C (71) and in four strains from clade A. The other 124 strains in the study were positive for this gene (Table S1). The other virulence related genes varied among the strains analyzed and no relation with host, year of isolation or clade were found (Table S1).

Twenty plasmid replicons were detected in the 199 *S.* Dublin strains studied, with a minimum of one and a maximum of six replicons found in a single isolate (Table S2). The most prevalent plasmid replicons were IncX1 and IncFII(S), found in respectively 197 and 177 out of the 199 strains studied (Table S2). Specifically, 11 plasmid replicons were detected in the 112 strains from Brazil in this study; two of them were exclusive from strains from Brazil, IncFII and Col440I. These plasmids are related to antimicrobial multidrug resistance genes and can be found in several *Enterobacteriales*^24,25^. However, the phenotypic antimicrobial resistance in the strains from Brazil were relatively low in comparison to the strains from other countries. Nine plasmid replicons were found only in strains isolated in other countries: IncFIB(S), IncFII (29), IncFII (pcoo), IncX4, IncI1-I (alpha), IncC, IncQ1, IncHI2, and IncHI2A (Table S2).

### Detection of antimicrobial resistance genes

All 199 *S*. Dublin strains studied presented at least two genes related to drug resistance: the *mdsA* and *mdsB* genes that encode an efflux pump (Table S3). In addition to the *mdsA* and *mdsB* genes, the strains from Brazil carried other resistance genes, such as *tet(A)* (n = 11), *tet(B)* (n = 4) and *tet(C)* (n = 4) related to tetracycline resistance, *blaTEM-1* (n = 4) related to beta-lactam resistance, *catA1* (n = 1) related to phenicol resistance, *aadA1* (n = 1) related to aminoglycoside resistance, and *sul1* (n = 1) related to sulfonamide resistance (Table S3). Sixteen strains presented point mutations in genes that might confer resistance to antimicrobials. Specifically, two strains presented the point mutation ramR_Y59H, related to tetracycline resistance, and the other 14 strains presented point mutations related to quinolone resistance: gyrA_D87N (n = 2), gyrA_D87G (n = 2), gyrA_S83F (n = 3), gyrA_S83Y (n = 2), gyrB_E466D (n = 1), and gyrB_S464F (n = 4) (Table S3).

Comparing the antimicrobial genotypic profiles and the phenotypic antimicrobial susceptibility of the strains from Brazil studied^15^, 21 out of 112 strains exhibited phenotypic resistance, and four strains (SD 4891, SD 747, SD 1847, and SD 484) had no correlation with the resistance genes found (Table S3).

### Multilocus Sequence Typing (MLST)

The 112 S. Dublin strains from Brazil presented nine different sequence types (STs) (Table S1). The STs found were ST 10 (n = 68), ST 3734 (n = 28), ST 4030 (n = 9), ST 4097 (n = 1), ST 4098 (n = 1), ST4100 (n = 2), ST 4101 (n = 1), ST 4232 (n = 1) and ST 4574 (n = 1). Specifically, ST3734 were found only in strains from Brazil belonging to clade A. The strains from other countries belonged to three different STs; ST 10 (n = 82), ST 2829 (n = 1), ST 4030 (n = 1), and no ST assigned (n = 3) (Table S1).

## Discussion

This study analyzed *S.* Dublin strains isolated over a 33 year-period in Brazil and contributed for a better characterization of an important bovine and human pathogen circulating in a country that is one of the largest beef producers and exporters worldwide^26^. The collected data were compared with those from 87 *S.* Dublin strains isolated from several countries and belonging to different SNP clades, recorded in the *Salmonella* database from the Pathogen Detection database (Fig. 1, Supplementary Table 1).

Whole genome SNP analysis divided the strains studied into three clades. The strains from Brazil were allocated into two specific clades A and C (Fig. 1, Supplementary Table 1). Specifically, clade A grouped strains from nine countries, including the reference *S.* Dublin strain SARB13 isolated in France in 1982, and 42 strains from Brazil. Thus, *S.* Dublin strains from clade A showed to be more diversified in comparison to the other clades, presenting strains isolated in several countries, mainly in Brazil, Europe, and the United States (Fig. 1, Supplementary Table 1).

Clade B comprised 25 strains isolated in UK (n = 16), Denmark (n = 2), Germany (n = 2), France (n = 1), Ireland (n = 1), and some strains from unidentified countries (n = 3). Clade C comprised the other 70 strains from Brazil and only one strain from the USA, suggesting that it is mainly a country-specific circulating subtype (Fig. 1, Supplementary Table 1).

The strains from Brazil isolated in nine States of the country did not clade according to the host or geography dependency. However, it was possible to note in the two cluster with Brazilian strains, some sub clusters with strains isolated either from humans or animals, isolated in similar periods and sharing similar gene repertoire (Fig. 1). This fact stress the zoonotic potential of *S.* Dublin circulating in Brazil for more than 30 years.

Analysis of the prevalence of *Salmonella* Dublin subtypes according to the country of isolation has identified that Brazilian strains belonged to two clades, and its major clade possibly shares a common ancestor with the UK strains^12^. This report corroborates the results from the present study that most strains from Brazil studied were allocated into two clades (Fig. 1, Supplementary Table 1).

Regarding virulence genes, all the strains studied, except SARB13 (SGSC-2470), did not carry any of the Vi antigen biosynthetic genes *tviA*, *tviB*, *tviC*, *tviD* and *tviE*, but all the strains carried SPI-7, where these genes are located (Fig. 1, Supplementary Table 1). All the strains also carried the *bstA* gene (*st313-td*); the degraded ST313-GI; the pathogenicity islands SPI-6 and SPI-19 that encode T6SS_SPI-6_ and T6SS_SPI-19_; the Gifsy-2 prophage that harbored an attachment and invasion protein; and the putative virulence factors *ggt* that encoded γ-glutamyl transpeptidase (GGT) and *pagN* that encoded an outer membrane protein (Fig. 1, Supplementary Table 1). The exception strain SARB13 carried all the Vi antigen biosynthetic genes but not the *bsta* gene (Fig. 1, Supplementary Table 1). These genes are highly prevalent in *S.* Dublin strains isolated all over the world^1^.

On the other hand, the frequency of some virulence genes in the Pathogen Detection database is clade- and country-specific. Interestingly, *iroB* and *iroC* genes that compose the siderophore Salmochellin were detected—both or one of them—, only in strains from Brazil, in 100 out of 112 strains from clades A and C (Fig. 1). Genes such as *cdtB* and *pltA*, related to a cytolethal distending toxin, *tibA*, an autotransporter related gene, *pilQ*, *pilR*, *pilV* and *pilW*, related to adherence (pili), and the genes *faeC*, *faeD* and *faeE*, related to adherence (fimbriae), presented a low prevalence and showed to be specific of some strains (Table S1). The *pegD* gene, related to fimbrial adherence determinants, were absent in all the strains from clade C (71) and in four strains from clade A. The other 124 strains in the study were positive for this gene (Table S1). The other virulence related genes searched varied among the strains in the study and no relation with host, year of isolation or clade were found (Table S1).

The plasmid replicons IncX1 and IncFII(S) were the two most prevalent in the set of strains studied (Table S2) and were related to the *S.* Dublin virulence plasmid pCT02021853_74 (Accession Number: NC_011204.1) that harbors the virulence gene *spv*^1^. These replicons are highly prevalent in *S.* Dublin strains independently of the country of isolation^1,11,12^. Two plasmid replicons were exclusively found in some strains from Brazil: (1) IncFII, similar to *E. coli* plasmid pC15-1A, was found in 23 strains—three from clade A and 20 from clade C; and (2) Col440I, from *Klebsiella pneumoniae* unnamed plasmid, was found in three strains, two from clade A and one from clade C (Table S2). The other plasmid replicons detected in the strains from Brazil were IncFIA and IncFIB (AP001918), similar to the *E. coli* plasmid k-12; IncFII (pRSB107), similar to the pRSB plasmid; and IncN, similar to the *S. Typhimurium* plasmid R46 and related to antimicrobial resistance^1,11,23,27^ (Table S2).

*S.* Dublin strains have high frequency of plasmids^1,11,12^. All the 197 *S.* Dublin strains isolated from cattle in Denmark carry virulence plasmids and an IncN plasmid related to a specific clade of the strains studied^11^. Kudirkiene and colleagues^11^ have reported for the first time the presence of IncFII and IncFIB replicons in *S.* Dublin strains, which are related to a specific clade of strains. In the present study, Brazilian *S.* Dublin strains from clades A and C carried IncN, IncFII, and IncFIB replicons (Table S2). The replicons IncX1and IncFII(S) are highly prevalent in strains isolated in several parts of the world, while the replicon IncA/C2, related to antimicrobial resistance, is exclusive to *S.* Dublin strains from the US^12^. In addition to the replicons IncX, IncFII, IncFIA, and IncFIB, Mohammed and colleagues^1^ have found the replicon IncQI, which was not detected in the strains examined in this study (Table S2).The different plasmid replicons found in the strains studied reinforced the diversity of *S.* Dublin strains in Brazil and in other parts of the world^1,11,12^.

In general, the *S.* Dublin strains from Brazil presented few antimicrobial resistance genes, corroborating literature reports on *S.* Dublin strains isolated in other parts of the world^1,11,12^. The presence of only one multidrug-resistant strain (SD 530; Table S3) reinforced this finding. The US strains are exceptions, with high number of antimicrobial resistance genes^12^.

The genes *mdsA* and *mdsB* were the most prevalent antimicrobial resistance genes detected in the strains studied (Table S3). These genes are part of the mdsABC complex, an efflux pump known to provide antimicrobial resistance to several drugs and toxins that is involved in *Salmonella* virulence and pathogenicity^28^. However, this study did not find a clear correlation between the presence of these two genes and a specific phenotypic resistance.

The other genes detected provided resistance to tetracyclines, beta-lactams, phenicols, aminoglycosides, and sulfonamides (Table S3). Twenty-three out of the 28 strains that carried any antimicrobial resistance gene correlated with the phenotypic resistance, demonstrating that WGS predicted antimicrobial resistance for *S.* Dublin strains in an accurate and reliable manner (Table S3).

Multilocus sequence typing analysis showed most of the strains studied belonging to ST 10, the main ST of serovar Dublin (Table S1). This sequence type was more prevalent in strains from other countries, 82 (94.25%) out of 87 strains, than in strains from Brazil, 68 (60.72%) out of 112 strains. The other STs found for strains from Brazil were single or double locus variant from ST 10, belonging to the same clonal complex. Specifically, ST3734 were found only in strains from Brazil belonging to clade A, which also comprised strains from other STs. The sequence types did not correlate with the sources and years of isolation of the strains in this study.

In conclusion, *S.* Dublin strains isolated in Brazil presented some few unique genes not found in strains from other countries and were allocated into two distinct clades with strains of human and animal origin epidemiologically related. This fact stresses the zoonotic potential of *S.* Dublin circulating in Brazil for more than 30 years.

## Supplementary Information

Supplementary Information 1.

Supplementary Information 2.

Supplementary Information 3.

## Data Availability

The datasets generated and/or analyzed during the current study are available in the GenBank repository in the BioProject PRJNA186035. The accession numbers of each strain are available in Table S1 and in Campioni et al., 2018^16^.
